# Association between pretreatment haemoglobin levels and morphometric characteristics of the tumour, response to neoadjuvant treatment and long-term outcomes in patients with locally advanced rectal cancers

**DOI:** 10.1111/codi.12307

**Published:** 2013-10-24

**Authors:** A A Khan, M Klonizakis, A Shabaan, R Glynne-Jones

**Affiliations:** * Department of Gastrointestinal Research, Mount Vernon Cancer Treatment and Research CentreLondon, UK; † Faculty of Health and Wellbeing, Centre for Sports and Exercise Science, Sheffield Hallam UniversitySheffield, UK; ‡ Department of Clinical Oncology, Minia University HospitalAl-Minia, Egypt

**Keywords:** Rectal carcinoma, neoadjuvant, radiotherapy, chemoradiation, haemoglobin

## Abstract

**Aim:**

The study was carried out to investigate whether pretreatment haemoglobin (Hb) levels act as a biomarker in the management of patients with locally advanced rectal cancer.

**Method:**

We prospectively collected data on all patients within our cancer network with localized low rectal cancer treated with preoperative radiotherapy/chemoradiotherapy at Mount Vernon Centre for Cancer Treatment between March 1994 and July 2008. Pretreatment Hb level was assessed as an independent variable for the whole study sample and dichotomised at a value of 12 g/dl. A multivariate analysis of covariance (MANCOVA) was conducted on parameters that had significant association on univariate analysis of covariance (ANCOVA) and correlational (Kendall tau/Pearson) analyses. Kaplan–Meier survival analysis and Cox proportional hazard models were used to determine significant prognostic markers. Statistical significance was set at 0.05.

**Results:**

463 patients (male/female 2:1; median age = 66 years, interquartile range = 56.5–73.0) were included in the analysis. There was significant tumour response of T stage (*P* < 0.001) and N stage (*P* < 0.001), with 17.6% of patients achieving a pathological complete response. Pretreatment Hb value was inversely related to the craniocaudal vertical tumour length (*P* = 0.02) and pretreatment T stage of the tumour (*P* = 0.01). Patients with Hb levels of < 12 g/dl and moderately differentiated adenocarcinoma were less responsive. Local recurrence was more common in patients with a pretreatment Hb of < 12 g/dl (hazard ratio = 1.78) over a median follow up of 24 months, but this was not statistically significant (*P* = 0.08).

**Conclusion:**

The pretreatment Hb level might be used as a biomarker of rectal tumour morphology, response to neoadjuvant chemoradiation and risk of local recurrence.

## Introduction

Locally advanced rectal cancer (LARC) has a high risk of local recurrence and distant metastases. The ability to identify patients at a higher risk of local failure using clinical, genetic and histopathogical biomarkers might allow individualized treatment and offer more effective and less-toxic treatments. However, biomarkers and tumour response to treatment have rarely been tested prospectively in large randomized studies and have not entered everyday clinical practice 1.

The presence of anaemia at the time of diagnosis, or its development during the course of neoadjuvant treatment, has been shown to be of prognostic value in patients with curable rectal cancer 2–5. Anaemia is a common presenting feature of patients with colorectal cancers 6 and may be associated with a more aggressive tumour behaviour and radioresistance 7. The ‘low oxygen tension’ within the tumour bed promotes angiogenesis and the formation of mutated tumour-cell lines that are capable of surviving at low levels of oxygen 8. In addition, chemoradiotherapy is also less effective because the cytotoxic effect of ionizing radiation and some chemotherapeutics are dependent on the formation of oxygen radicals 9.

The presence of anaemia in patients with newly diagnosed rectal cancer may indicate a larger tumour with advanced disease or an inherent feature of biologically aggressive behaviour. The haemoglobin (Hb) level at diagnosis may provide insight into rectal tumour morphology and biology. To our knowledge, this hypothesis has not been previously tested.

The aim of the present study was to investigate whether Hb levels at diagnosis can be used (i) as a biomarker of rectal tumour morphology and hence reflect the stage of disease, (ii) to predict rectal cancer response to neoadjuvant treatment, and (iii) to determine long-term cancer specific outcome.

## Method

### Patients

We analysed data on all consecutive patients from our prospectively collected database. All patients had histologically proven adenocarcinoma of the rectum and were planned for curative treatment with neoadjuvant radiotherapy, with or without concomitant chemotherapy, at the Mount Vernon Cancer Treatment and Research Centre between March 1994 and December 2008. The data collected are presented in Tables 1 and 2.

**Table 1:** Data collected and descriptive statistics.

Characteristic	Value
Gender, *n* (%)	
Female	149 (32.2)
Male	314 (67.8)
Age (years)	
Median	66
IQR	56.5–73.0
ASA grading, *n* (%)	
1	28 (12.6)
2	141 (63.2)
3	53 (23.8)
4	1 (0.4)
Haemoglobin level (g/dl)	
Mean	12.96
SD	1.8
Range	8.2–19.5
CEA level (μg/l)	
Median	4
IQR	2–10
Tumour characteristics, *n* (%)	
Primary	443 (96.1)
Recurrent	18 (3.9)
Distance from anal verge (cm)	
Mean	5.07
SD	2.7
Range	0–18
Tumour length (cm)	
Mean	5.78
SD	2.2
Range	0–14
Tumour staging, *n* (%)	
T2	26 (6.1)
T3	219 (51.5)
T4	180 (42.4)
N0	147 (35.0)
N1	156 (37.1)
N2	116 (27.6)
N3	1 (0.2)
M0	381 (93.8)
M1	25 (6.2)
Type of operation, *n* (%)	
Anterior resection	154 (33.3)
APER	232 (49.9)
Hartmann’s	4 (0.9)
Not resectable	65 (14.0)
Refused surgery	8 (1.7)
Postoperative complications, *n* (%)	
No	248 (63.3)
Yes	144 (36.7)

APER, abdominoperineal excision of the rectum; ASA, American Society of Anesthesiology; IQR, interquartile range.

**Table 2:** Different neoadjuvant chemotherapy regimes for the study group.

Chemotherapy regime	Number of patients
No chemotherapy	54
5-FU	3
5-FU (BOSSET regime)	189
5-FU + folinic acid	18
5-FU + leucovorin	7
5-FU + oxaliplatin	1
5-FU + oxaliplatin + folinic acid	1
Capecitabine	124
Capecitabine + oxaliplatin	3
Cetuximab	2
CORE trial	6
De Gramont regime	3
DESCARTES	11
Mitomycin	1
Palliative chemotherapy	1
SOCRATES	22
Tomudex	1
Tomudex + oxaliplatin	1
XELOX trial	15
Total	463

5-FU, 5-fluorouracil; BOSSET, regime with different doses of 5-FU; CORE trial, Phase II study – Chemoradiation with Oxaliplatin in Rectal Cancer; de Gramont regime, regime using fluorouracil and folinic acid; DESCARTES, regime using irinotecan in the chemoradiation; SOCRATES, Phase II study – oxaliplatin, capecitabine and radiotherapy; XELOX, capecitabine and oxaliplatin.

### Definition of parameters

We conducted the analysis using *‘*Hb level’ as a continuous variable and dichotomised the study population for further analysis at the Hb value of 12 g/dl. This Hb level cut-off value was based on the findings of Vaupel 8,10 who showed that Hb levels of < 12 g/dl result in significant tumour hypoxia, thus altering its behaviour. The same cut-off value has been used to define anaemia in previous studies on this topic 2. ‘Tumour morphology’ was defined by pretreatment endoluminal tumour length, circumferential nature, distance from the anal verge, differentiation and T stage and pathophysiological attributes such as lymph node involvement, distant spread and carcinoembryonic antigen (CEA) levels. The ‘nRT regime’ was either short-course (SCPRT: 20 Gy in five daily fractions) or long-course (LCRT: 45 Gy in 25 fractions over 5 weeks), either alone or with fluoropyrimidine-based regime*,* with a few patients receiving additional cytotoxic drugs (Table 2). Patients receiving SCPRT were only included in the analysis for local recurrence. ‘Downstaging’ was defined by comparing the most advanced radiological stage (cTNM) with the histological stage (ypTNM). Consultant colorectal surgeons claimed to undertake standard ‘total mesorectal excision (TME) surgery’, but we have no data on the quality of the mesorectal excision 11. ‘Pathological complete response’ was defined as no evidence of malignant cells on microscopic examination (although no standardized procedure was performed 12. Patients were followed for a median of 24 months and 230 for at least 21 months.

### Statistical analysis

For the purpose of this report, univariate and multivariate analyses were carried out for the entire population and populations in each Hb group. Failures for the efficacy end-points were as follows: overall survival: death from any cause; and disease-free survival: locoregional failure, distant failure or death from any cause. All end-points were assessed from the start of treatment to the date of first failure for the given end-point or the date of the last follow up for patients who did not fail at a given end-point.

Initial statistical analysis explored baseline correlations between independent variables from the data set (Table 1) and study end-points using Kendall’s tau and Pearson tests. The strength of any correlation was further examined by univariate analysis of covariance (ANCOVA) followed by multivariate analysis of covariance (MANCOVA). The Wilcoxon signed-rank test was used to assess tumour down-staging. Survival analyses were performed using the Kaplan–Meier method and comparisons were tested using the log-rank test. Backward stepwise (likelihood ratio) Cox-regression survival analysis was used to establish a model displaying any relationships between chosen variables and outcomes. Statistical significance was set at *P* < 0.05. The data were analysed using SPSS 19 (IBM, USA).

## Results

### Descriptive results

A total of 463 patients (male/female ratio = 2:1; median age = 66 years; interquartile range (IQR) = 56.5–73.0) were included in the analysis (Table 1). Twenty-seven (0.05%) patients received SCPRT only and the remainder received LCRT with concomitant chemotherapy. In the LCRT group, the median interval to surgery was 69 (IQR = 55–83) days. There was significant tumour response of T stage [number of patients who responded (mean rank) = 170 (94.25); number of patients with progression (mean rank) = 12 (52.2); *P* < 0.001] and N stage [number of patients who responded (mean rank) = 140 (88.43); number of patients with progression (mean rank) = 32 (78.08); *P* < 0.001] regression, with 17.6% of patients achieving a pathological complete response. In 65 (14%) patients the tumour was unresectable, and eight (1.7%) patients refused surgery.

### Hb *vs* morphometric tumour characteristics

The pretreatment Hb level had a significant, inverse relationship with tumour length (univariate *P* = 0.002; multivariate *P* = 0.02) and pretreatment clinical T stage (*P* = 0.01). There was no association between Hb levels and clinical N stage (*P* = 0.8), distance from the anal verge (*P* = 0.68) and baseline CEA values (*P* = 0.8).

### Hb *vs* response to neoadjuvant treatment

As a continuous variable, the pretreatment Hb level did not show any association with tumour response; however, with dichotomisation with a cut-off value of 12.0 g/dl, patients with Hb < 12 g/dl showed less response to neoadjuvant chemoradiation, if regression of T stage was considered as a response (*P* = 0.03). Patients with Hb ≥ 12 g/dl showed a strong association with a pathological complete response in univariate analysis (*P* = 0.02); however, this association was lost in multivariate analysis.

### Hb *vs* long-term prognosis

**Figure 1:**
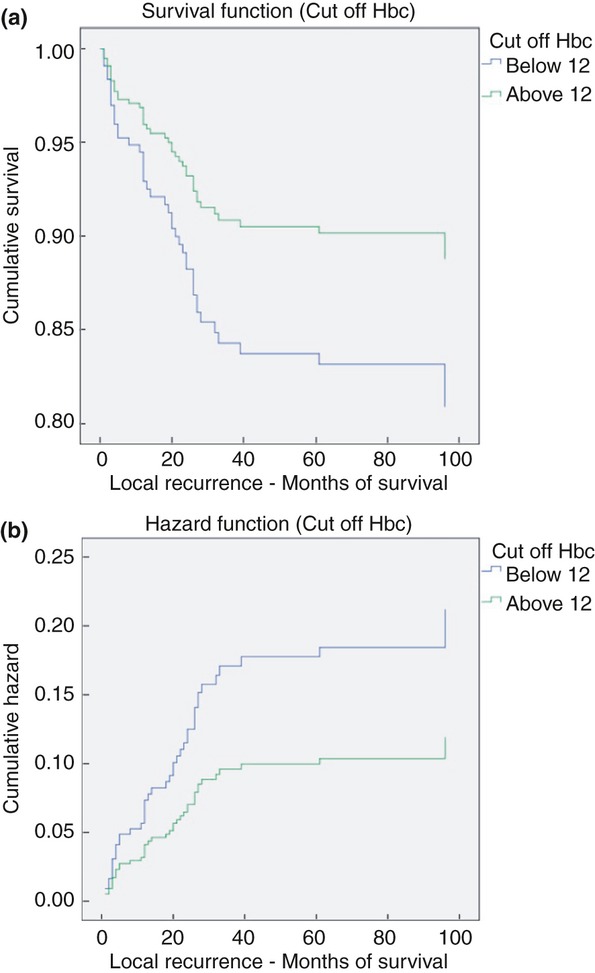
Kaplan–Meier curves showing a statistically significant difference in the frequency (a) and risk (b) of local recurrence between patients with a baseline haemoglobin (Hb) of less (blue line) or more (green line) than 12 g/dl.

Over a median follow-up period of 24 (1–95) months, the crude local recurrence rate was 20.3% and the 3-year actuarial local recurrence was 8%. The statistical model used to determine risk factors of local recurrence showed that pretreatment Hb < 12g/dl (hazard ratio > 1; Fig. 1 and Table 3), higher pretreatment T stage, shorter distance from the anal verge and high baseline CEA value increase the risk of local recurrence. The influence of Hb < 12 g/dl on local recurrence was not statistically significant (*P* = 0.08). We did not find a relationship between Hb level and distant metastasis or overall survival.

**Table 3:** Final parameters with significant influence on local recurrence in 3 years.

Risk factors	Hazard ratio	*P*-value
Distance from anal verge (cm)	0.86	0.03
CEA value	1.00	0.01
Operation type	2.07	0.00
Distant metastasis	6.45	0.00
Cut-off Hb levels (12 g/dl)	1.78	0.08
Tumour length (cm)	1.17	0.01

CEA, carcinoembryonic antigen.

Note that patients with a haemoglobin (Hb) of < 12 g/dl are at risk of developing local recurrence (hazard ratio = 1.78) but this was not statistically significant (*P* = 0.08).

## Discussion

Pretreatment anaemia has shown significant prognostic value in solid cancers, including tumours of the head and neck 13, breast 14, oesophagus 15, stomach 16, uterine cervix 17 and bladder 18. Intraluminal bleeding from a friable endoluminal tumour surface can result in substantial blood loss and therefore the degree of anaemia is commonly severe in gastrointestinal tumours compared with anaemia as a paraneoplastic phenomenon in other malignancies. About 30% of patients with rectal cancer present with anaemia 19. The presence of pretreatment anaemia in rectal cancer patients is associated with a worse tumour response 4, locoregional control 3, disease-free survival 5 and overall survival 2. Our results support the association of pretreatment Hb with tumour response and locoregional control. Moreover, our analysis showed that non-anaemic patients were more likely to achieve a pathological complete response in univariate analysis.

Morphometric characteristics of tumour in resected specimens have been shown to be independent prognostic markers in early-stage cancer of the uterine cervix 20 and predictors of positive lymph nodes in colorectal cancers 21. In rectal cancer, pretreatment tumour size has been shown to influence the outcome of laparoscopic surgery 22. Therefore, pretreatment tumour length may have a prognostic role. Our study demonstrates that the pretreatment Hb level might reflect endoluminal tumour length and pretreatment clinical T stage. Patients with larger tumours had significantly lower Hb levels.

The presence of anaemia is related to aggressive tumour behaviour and poor prognosis through exacerbating tumour hypoxia 23. There are several biological factors that can explain how anaemia can lead to a poor outcome. Evidence from studies on cancer of the head and neck 24 and the uterine cervix 25 and on soft tissue sarcomas 26 clearly demonstrate the adverse prognostic impact of tumour hypoxia. Within the tumour bed, structural and functional abnormalities result in haphazard architecture, hindering oxygen diffusion from vessels to individual cells and causing hypoxia. Low oxygen tension within cancer cells instigates biological behaviours that lead to a more aggressive phenotype and the induction of angiogenesis. High levels of circulating angiogenic factors have also been reported in patients with anaemia 27. Furthermore, the effectiveness of radiotherapy is dependent on the generation of free oxygen radicals that induce DNA and membrane damage within cancer cells 28. Lack of oxygen within tissue hinders free-radical generation, hence creating resistance to treatment. Tumour hypoxia also seems to diminish the efficacy of certain chemo- and immunotherapeutics that are dependent on normal local oxygen levels.

Correction of pretreatment anaemia provides symptomatic relief but without any effect on tumour behaviour 29. Homologous blood transfusion has been shown to increase infective complications as a result of immunosuppressive effects, especially if given perioperatively 30. Erythropoiesis-stimulating agents (ESA) have been shown to counteract the effects of cancer-induced anaemia, but a recent meta-analysis of randomized controlled trials did not show any significant effect on survival and disease progression. There is also an added risk of thromboembolic complications 31. However, treatment with ESA has not been tried in rectal cancer 32. Hence, anaemia may be an inherent feature of tumours with aggressive potential, and the pretreatment Hb level may be simply a marker of such behaviour.

One weakness of the study was the accuracy of the measurement of tumour length. Preoperatively this is observer dependent and therefore repeatability analysis of this parameter should be included in future studies. Another limitation of this study was that not all surgeons were performing TME in the 1990s and therefore the quality of surgery was variable.

We propose risk categorization 33 of patients presenting with rectal cancer as a possible way of individualizing management. Anaemia should be considered as a potential risk factor associated with poor tumour morphology, more aggressive behaviour and worse long-term prognosis. Measurement of Hb is simple and cheap and may distinguish rectal cancer subtypes with distinct clinical courses. The use of Hb level may improve decision-making, and perhaps combinations of factors such as Hb level, tumour length and T stage may, in future, allow definition of distinct subgroups with differing clinical biology.

In conclusion, we have shown that the pretreatment Hb level provides information on the nature of the rectal cancer and its potential behaviour in resistance to treatment and the chance of local recurrence. Our analysis revealed Hb to be a clinically applicable biomarker, which may be useful in large prospective trials to assess risk-adapted therapies. Absolute and cut-off values of Hb could easily and reliably be used between different laboratories to classify high- and low-risk groups.
